# Homocysteine Induces Hepatic Steatosis Involving ER Stress Response in High Methionine Diet-Fed Mice

**DOI:** 10.3390/nu9040346

**Published:** 2017-04-01

**Authors:** Yanbiao Ai, Zhenzhen Sun, Chuan Peng, Lingli Liu, Xiaoqiu Xiao, Jibin Li

**Affiliations:** 1 Department of Nutrition and Food Hygiene, School of Public Health and Management, Chongqing Medical University, Chongqing 400016, China; aiyanbiao1992@163.com (Y.A.); lipglucolabszz@163.com (Z.S.); liulingli0_0@163.com (L.L.); 2 Laboratory of Lipid & Glucose Metabolism, The First Affiliated Hospital of Chongqing Medical University, Chongqing 400016, China; 13527441813@163.com

**Keywords:** homocysteine, methionine, ER stress, NAFLD

## Abstract

Elevated circulating homocysteine (Hcy) has been proposed to be associated with non-alcoholic fatty liver disease (NAFLD). It is also reported that Hcy causes protein misfolding in the endoplasmic reticulum (ER). In this study, we used a high methionine diet (HMD)-fed mouse model and cultured primary hepatocytes to investigate the effects of Hcy on hepatic lipids metabolism. C57BL/6J mice received either standard chow diet (CT, *n* = 10) or diet supplemented with 2% methionine (MET, *n* = 10) for 16 weeks. In in vitro experiments, cultured mouse primary hepatocytes were treated with Hcy, or Hcy combined with 4-phenylbutyric acid (4-PBA), or tunicamycin (TM), respectively. HMD-fed mice exhibited a mild increase in plasma Hcy level. There was no significant difference of body weight gain between the two groups. Nevertheless, HMD feeding increased epididymal fat/body weight ratio, elevated plasma triglyceride (TG) level, and decreased high-density lipoprotein cholesterol (HDL) level. Similarly, mice on HMD displayed higher liver/body weight ratio, plasma aspartate aminotransferase (AST) and its ratio to alanine aminotransferase (ALT), which was supported by the morphological observations of hepatic triglyceride accumulation in liver tissue as well as primary hepatocytes. Activation of the sterol response element-binding protein 1c (SREBP1c) in Hcy-treated hepatocytes with increased expression of genes involved in hepatic de novo lipogenesis was partially reduced by pretreatment of 4-PBA. Hcy-induced ER stress was also ameliorated by 4-PBA pretreatment, thus demonstrating an important role of Hcy-induced ER stress response in hepatic steatosis. These findings suggest that elevated Hcy was a critical factor in the pathogenesis of NAFLD. Activation of the ER stress response may be involved in Hcy-induced hepatic steatosis.

## 1. Introduction

Nonalcoholic fatty liver disease (NAFLD) has become a serious global health issue. It is estimated that 25% of the adult population in the world are affected [1]. NAFLD is characterized by the accumulation of triglyceride in the liver, which presents a wide spectrum of hepatic metabolic disorders ranging from simple steatosis to inflammatory steatohepatitis (NASH) and cirrhosis [2]. NAFLD is strongly associated with obesity, type 2 diabetes, dyslipidemia, and other metabolic disorders, including hyperhomocysteinemia [1,3]. Homocysteine (Hcy) is an intermediate produced from the demethylation of methionine. Hcy is removed through two major pathways: the remethylation reaction and the transsulfuration reaction [4,5,6]. Elevated Hcy in the blood is associated with dietary nutritional factors such as low folate, vitamin B_6_, vitamin B_12_, and excess methionine intakes [7,8] and genetic abnormality [9]. Recently, hyperhomocysteinemia has been implicated in a variety of diseases, including cardiovascular disease, diabetes, and hepatic steatosis [10,11,12,13,14].

The endoplasmic reticulum (ER) plays an important role in regulating protein synthesis, lipid metabolism, and calcium homeostasis [15,16]. Increasing evidence suggests that the ER stress response plays an important role in NAFLD development. The activated unfolded protein response (UPR) in the liver can be observed in a variety of diet-induced or genetic modification diseases in animal models [17,18]. Meanwhile, the ER stress response in a variety of cells such as hepatocytes and endothelial cells can be induced by elevated levels of Hcy [19,20]. Most of the studies on the effects of Hcy in animal models or cultured cells adopted high levels. Therefore, in the current study, we used a diet-induced animal model coupled with high Hcy treatment of primary hepatocytes to investigate the effects of elevated Hcy level on liver lipids metabolism and the possible role of ER stress response during the development of hepatic steatosis.

## 2. Materials and Methods

### 2.1. Animals and Experimenalt Protocols

Twenty male C57BL/6J mice at 4 weeks of age were maintained in 12-h light/dark cycles in the animal facilities of Chongqing Medical University with unlimited access to food and water. After 2 weeks of feeding a standard chow, mice were divided randomly into a control group (CT, *n* = 10) and a high methionine diet (HMD, *n* = 10) group. The mice in the CT group remained on standard chow, and those of the HMD group were given the same diet but supplemented with 2% methionine (MET) for 16 weeks. Body weights were monitored every week throughout the study. At the end of the dietary treatment, all mice were anesthetized with ether after a 12-h fast. Whole blood samples collected via the retro-orbital sinus were centrifuged at 2000× *g* for 10 min at 4 °C to obtain plasma. The liver and epididymal fat were removed, weighed, immediately frozen in liquid nitrogen, and then stored at −80 °C until use. The animal study was approved by the Ethics Committee of Chongqing Medical University (2015-3-5).

### 2.2. Biochemical Analysis

Hcy concentrations were measured by mouse Hcy ELISA kit (Yanhui, Shanghai, China, Lot. No. CK-E-91626M). Plasma levels of triglyceride (TG), cholesterol, alanine aminotransferase (ALT), aspartate aminotransferase (AST), high-density lipoprotein cholesterol (HDL) and low-density lipoprotein cholesterol (LDL) were measured using an automated biochemical analyzer (Siemens ADVIA^®^ 2400 Chemistry System, Tarrytown, NY, USA).

### 2.3. Liver Histology

The liver was excised from each mouse after sacrifice, fixed in 4% paraformaldehyde in 0.1 mol/L phosphate-buffered saline (PBS; pH = 7.4), and embedded in paraffin for staining with hematoxylin and eosin (H & E). The stained slices were examined using Plus Image-Pro 6 image analysis system (American Cybernetics Media Company, Rockville, MD, USA) at 200× magnification.

### 2.4. Primary Hepatocyte Culture and Experimental Protocols

Hepatocytes were isolated from 8-week male C57/6J mice by two-step perfusion method as described previously [21]. Briefly, the liver was perfused with calcium-free perfusion buffer medium through the portal vein until the liver changed to pale in color, and was then changed to perfusion with digestion buffer medium. The hepatocytes were obtained by centrifuging the medium twice and maintaining in 10% fetal bovine serum (FBS) (Gibco^®^, South Melbourne, Victoria, Australia) Dulbecco’s Modified Eagle Medium/Nutrient Mixture F-12 (DMEM/F12) medium (Gibco^®^, Shanghai, China) supplemented with 100 units/mL penicillin, 100 µg/mL streptomycin, 10 μg/mL insulin, 0.1 µmol/L dexamethasone, and 5 ng/mL epidermal growth factor. Before the experiment, the hepatocytes were treated with serum-free DMEM/F12 medium containing either 5 mmol/L Hcy, 5 mmol/L methionine, 2 mmol/L 4-phyenylbutyric acid (4-PBA), or 2 μg/mL tunicamycin (TM), respectively, for 24 h.

### 2.5. Oil Red O Staining

The cultured hepatocytes grown on glass coverslip were washed with PBS three times and then fixed with 4% paraformaldehyde for 40 min. The fixed cells were washed with 60% isopropanol for 8 s and then PBS three times. In darkness, the cells were stained with freshly diluted Oil Red O working solution for 1 h, and counterstained with hematoxylin for 3 min. The cells were washed with PBS three times and then observed using a microscope.

### 2.6. Real Time Quantitative RT-PCR

Total RNA was extracted from liver samples and cultured primary hepatocytes using Tripure Isolation Reagent (Roche, Mannheim, Germany) according to the manufacturer’s instructions. cDNA was synthesized by using the Reverse Transcription Kit (TaKaRa, Otsu, Japan). Real-time quantitative PCR analysis was performed with SYBR Green in a thermal Cycler Dice Real Time System (TaKaRa, Otsu, Japan). The relative mRNA levels of target genes were analyzed by using the 2^−ΔΔCt^ method. Each sample determination was repeated three times. The sequences of primer pairs (forward and reverse, respectively) were as follows: β-actin: 5′-TGCTGTCCCTGTATGCCTCTG-3′ and 5′-TCTTTGATGTCACGCACGATTT-3′, BHMT: 5′-AGCATCCTGAGGCAGTTCGT-3′ and 5′-TCTGCATGACGTTCGATCCA-3′, CBS: 5′-CAGCACCCTCCCCTCCTAA-3′ and 5′-GTAAGCTACTCGGGCATAGAGGAT-3′, GRP78: 5′-CAGGGCAACCGCATCAC-3′ and 5′-CAATCAGACGCTCCCCTTCA-3′, CHOP: 5′-GCATGAAGGAGAAGGAGCAG-3′ and 5′-CTTCCGGAGAGACAGACAGG-3′, IPPi: 5′-GGCGAGCTGGAAGAGAACAA-3′ and 5′-TTCCCAACTCGGCTTTCAAG-3′, FPPs: 5′-CCCTGCCCCCATCCA-3′ and 5′-GGGTCACTTTCTCCGTTTGTAGA-3′, HMG-CoAr: 5′-CCAAACCCCGTAACCCAAA-3′ and 5′-CGACTATGAGCGTGAACAAGGA-3′, ACC1α: 5′-GTTTCAGAACGGCCACTACGA-3′ and 5′-CATTGTCACCAGGAGATTCTTTTTG-3′, FAS: 5′-GGCACTGACTGTCTGTTTTCCA-3′ and 5′-GTAAAAATGACACAGTCCAGACACTTC-3′.

### 2.7. Westaern Blotting

Total protein was isolated from the liver homogenates and cultured primary hepatocytes with RIPA (radioimmune protection assay) lysis buffer, and protein concentrations were determined with the BCA kit (P0012, Beyotime Biotechnology, Jiangsu, China) according to the manufacturer’s instructions. Protein of each sample (40 μg) was separated by 8% sodium dodecyl sulfate polyacrylamide gel electrophoresis (SDS-PAGE) and electro-transferred onto the polyvinylidene difluoride (PVDF) membranes. Then, the PVDF membranes were blocked with 5% bovine serum albumin (BSA) for 1 h at room temperature and incubated with primary antibodies (1:1000) overnight at 4 °C, and then incubated with the secondary antibodies (1:5000) for 1 h. the membranes were washed with phosphate-buffered saline with tween-20 (PBST; pH = 7.4) three times for 5 min. The immunoreactive bands were visualized using an enhanced chemiluminescence (ECL) detection system. The protein band densities were quantified with Fusion software.

### 2.8. Statistical Analysis

Data were expressed as mean ± SD. Statistical analyses were performed with independent *t*-test, one-way ANOVA, followed by Fisher’s least significant difference (LSD’s) multiple comparison test using SPSS 18.0 analysis software (SPSS, Chicago, IL, USA). *P*-values less than 0.05 were considered statistically significant.

## 3. Results

### 3.1. HMD Feeding Induced Hepatic Steatosis in Mice

**Figure 1:**
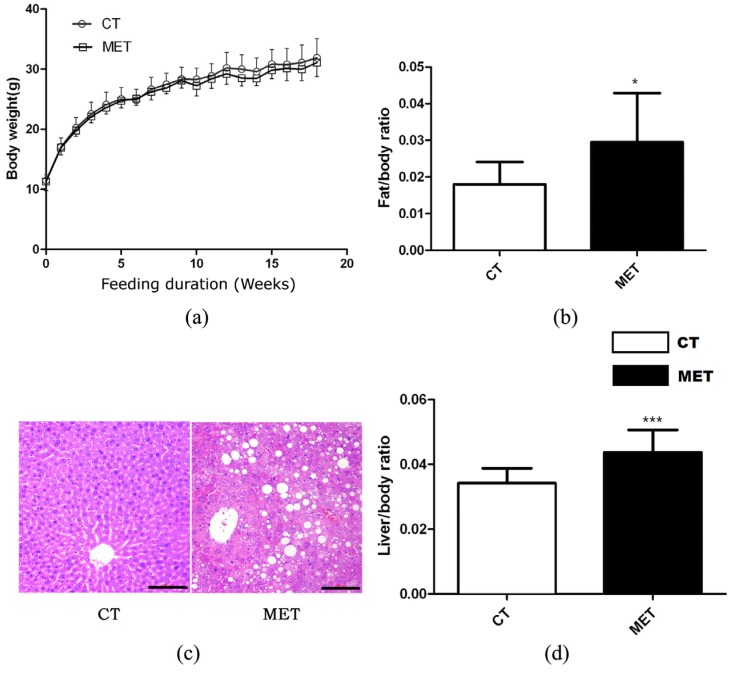
Effects of high methionine diet (HMD) feeding on: (**a**) Body weight gain; (**b**) Epididymal fat/body weight ratio; (**c**) Hematoxylin and eosin (H & E) staining of the liver (magnification 200×), scale bars, 100 μM; (**d**) Liver/body weight ratio. Data are presented as mean ± SD. * *p* < 0.05 and *** *p* < 0.001 vs. control (CT). CT: standard chow diet; MET: diet supplemented with 2% methionine.

High methionine diet feeding did not result in any significant effects on body weight or absolute liver weight of mice in different periods (Figure 1a). However, HMD feeding induced significant increases in liver/body weight ratio (*p* < 0.01) and epididymal fat/body weight ratio (*p* < 0.05) (Figure 1b,d). Accordingly, H & E staining of liver sections confirmed the presence of hepatic lipid accumulation in the MET group after 16 weeks HMD feeding (Figure 1c). Mice in the MET group exhibited increased levels of TG, AST, AST/ALT, but decreased HDL level. Nevertheless, the two groups did not differ in TC, ALT, or LDL levels (Table 1).

### 3.2. HMD Feeding Increased Plasma Hcy Level in Mice

**Figure 2:**
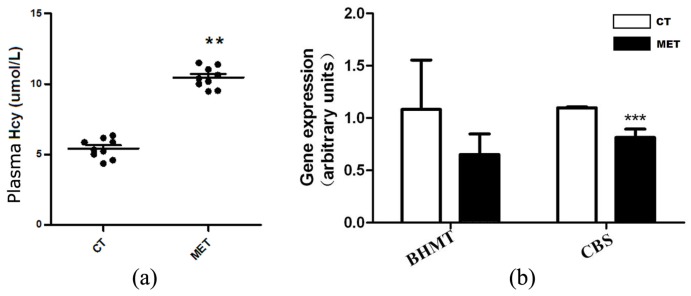
HMD feeding elevated plasma homocysteine (Hcy) level. (**a**) Plasma Hcy levels in mice fed with chow or high methionine diets; (**b**) The mRNA expression levels of betaine-methyltransferase (BHMT) and cystathionine-β-synthase (CBS) in the liver. Data are presented as mean ± SD. ** *p* < 0.015 and *** *p* < 0.001 vs. CT group.

Plasma Hcy level was elevated in the MET group (10.47 ± 0.75 μmol/L) compared with the CT group (5.36 ± 0.67 μmol/L, *p* < 0.015) (Figure 2a). We further examined the relative expression levels of two genes involved in Hcy metabolism. HMD feeding significantly down-regulated cystathionine-β-synthase (CBS) mRNA level (*p* < 0.01), but not betaine-methyltransferase (BHMT) (Figure 2b).

### 3.3. Hcy Increased Hepatic Lipogenesis

**Figure 3:**
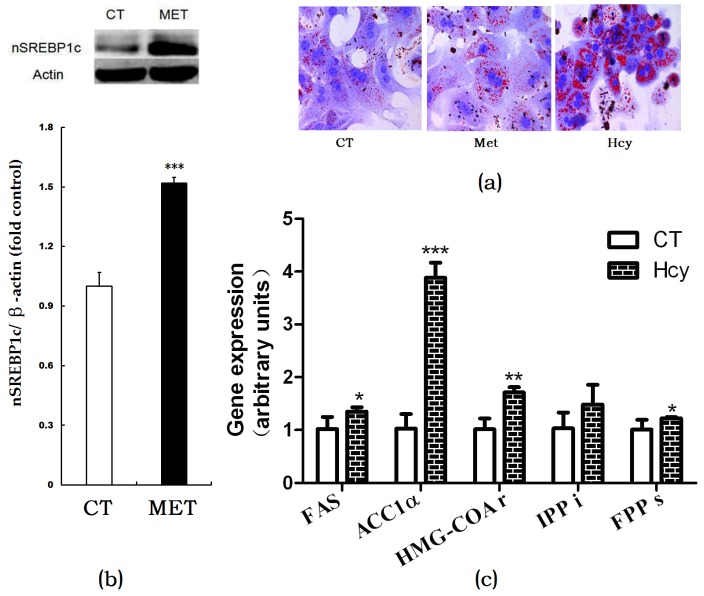
Effects of Hcy on hepatic lipogenesis. (**a**) Oil Red O staining of cultured primary hepatocytes treated with methionine or Hcy; (**b**) Protein expression of nSREBP1c in the liver; (**c**) Relative expression of genes involved in hepatic lipogenesis. The data are presented as mean ± SD. * *p* < 0.05, ** *p* < 0.015, and *** *p* < 0.001 vs. CT. FAS: fatty acid synthase; ACC1α: acetyl-CoA carboxylase 1α; HMG-COAr: hydroxymethylglutaryl CoA reductase; IPPi: isopentenylpyrophosphate isomerase; FPPs: farnesyl diphosphate synthase.

Oil Red O staining of cultured primary hepatocytes indicated that Hcy treatment increased hepatic accumulation compared with the CT or methionine-treated cells (Figure 3a). In HMD-fed mice, the nSREBP1c expression level in the liver was significantly higher than that of control (Figure 3b). Accordingly, mRNA expression levels of hepatic de novo lipogenesis (DNL)-related genes such as FAS, ACC1α, HMG-CoAr, FPPs in Hcy-treated primary hepatocytes were increased compared with those in control group cells (Figure 3c).

### 3.4. ER Stress Response-Mediated Hcy-Induced Hepatic Steatosis

**Figure 4:**
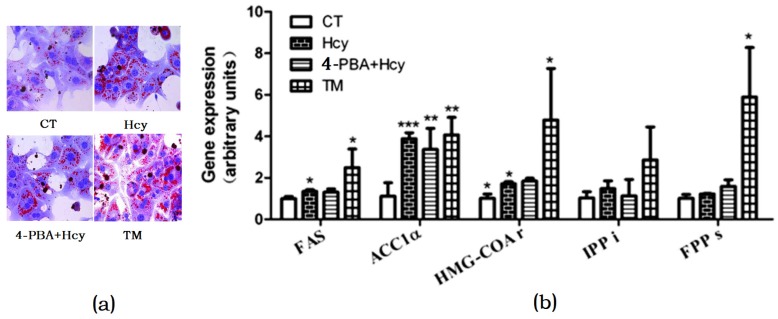
Endoplasmic reticulum (ER) stress response involved in hepatic steatosis. (**a**) Oil Red O staining of cultured primary hepatocytes; (**b**) Relative expression of genes involved in hepatic de novo lipogenesis. The data are presented as mean ± SD. * *p* < 0.05, ** *p* < 0.015, and *** *p* < 0.001 vs. CT. 4-PBA: 4-phyenylbutyric acid; TM: tunicamycin. FAS: fatty acid synthase; ACC1α: acetyl-CoA carboxylase 1α; HMG-COAr: hydroxymethylglutaryl CoA reductase; IPPi: isopentenylpyrophosphate isomerase; FPPs: farnesyl diphosphate synthase.

To investigate whether Hcy triggered ER stress in hepatocytes, cells were treated with 2 µg/mL ER stress inducer tunicamycin (TM) or pretreated with 2 mM ER stress inhibitor 4-Phenylbutyric acid (4-PBA) for 1 h, and then incubated with 5 mM Hcy (4-PBA + Hcy) for 24 h. Oil Red O staining of hepatocytes treated with Hcy or TM revealed that there was increased TG accumulation compared with control group. In contrast, pretreatment with 4-PBA decreased Hcy-induced TG accumulation in hepatocytes (Figure 4a). Accordingly, compared with the control group, Hcy and TM treatment increased the mRNA levels of FAS, ACC1α, and HMG-CoAr. However, pretreatment with 4-PBA prevented these changes (Figure 4b).

**Figure 5:**
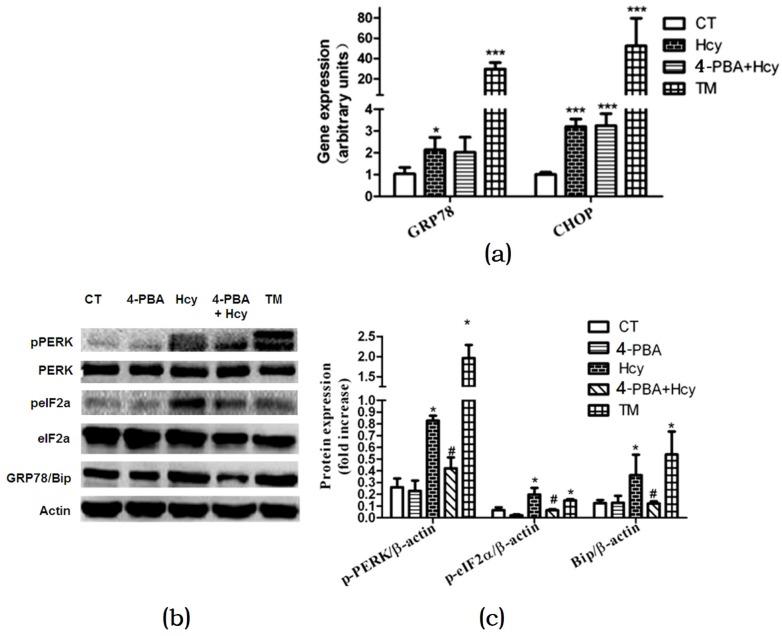
Effects of Hcy on ER stress response in primary hepatocytes: (**a**) Relative expression of genes of ER stress biomarkers; (**b**,**c**) Relative expression of proteins involved in ER stress pathway in primary hepatocytes. The data are presented as mean ± SD. * *p* < 0.05, ** *p* < 0.015 vs. CT, # *p* < 0.05 vs. Hcy and TM. GRP78: glucose regulated protein 78; Bip: binding immunoglobulin protein; CHOP: c/EBP homologous protein; p-PERK: phosphorylated protein kinase RNA-like ER kinase; p-eIF2α: phosphorylated eukaryotic initiation factor 2α.

To further validate the role of the ER stress response in Hcy-induced hepatic steatosis, we investigated changes in ER stress markers. Hcy or TM treatment increased glucose regulated protein 78 (GRP78) and c/EBP homologous protein (CHOP) mRNA expression levels compared with control (Figure 5a). Nevertheless, 4-PBA pretreatment down-regulated mRNA expression level of GRP78 but not CHOP in hepatocytes treated with Hcy (Figure 5a). We further detected the impact of Hcy treatment on the protein kinase RNA-like ER kinase (PERK) signal pathway in primary hepatocytes. Compared with control, the protein levels of GRP78 and PERK, and eukaryotic initiation factor 2α (eIF2α) phosphorylation were significantly elevated in hepatocytes treated with ER stress-inducing agent TM. Consistent with these findings, GRP78, p-PERK, and p-eIF2α were also increased after Hcy treatment for 24 h. Besides, 4-PBA pretreatment down-regulated Hcy-induced elevated GRP78, p-PERK, and p-eIF2α (Figure 5b,c).

## 4. Discussion

Hyperhomocysteinemia is well known as an independent risk factor for cardiovascular disease, and has been proposed to be a potential factor for nonalcoholic fatty liver disease [5,6,7,22]. The major findings of our study were that plasma Hcy level could be elevated by modified diet without other interventions, and that diet-induced moderate hyperhomocysteinemia led to hepatic steatosis, and other altered plasma biochemical parameters as well. We further validated the role of ER stress response in Hcy-induced triglyceride accumulation in primary hepatocytes.

Hcy is an intermediate of methionine metabolism in the liver. In the present study, we adopted a moderate hyperhomocysteinemia animal model induced by HMD [23] rather than giving exogenous Hcy intravenously. Previous studies had successfully observed the pathophysiological outcome of hyperhomocysteinemia in vivo by using a high-methionine/low-folate diet or gene-modified animal models [24]. The plasma Hcy levels were usually more than 20 μM in these studies. In our diet-induced animal model, the plasma Hcy levels ranged from 9.72 to 11.22 μM, which were approximately twofold higher than those in control group (4.96–6.03 μM). The pathways for Hcy removal involve two reactions. The first pathway is remethylation to methionine catalyzed by betaine-homocysteine methyltransferase (BHMT) or methionine synthase (MS); the second pathway is transsulfuration reaction catalyzed by cystathionine β-synthase (CBS). In the current study, reduced mRNA relative expression level of CBS suggested that HMD-induced hyperhomocysteinemia might result from the blocking of the transsulfuration pathway. However, we did not detect the enzymatic activity of CBS in HMD-fed mice. Therefore, additional studies are necessary to determine whether this pathway is involved in HMD-induced Hcy alteration.

Interestingly, the moderately increased Hcy concentration resulted in elevated liver/body weight ratio, plasma AST, and TG, and reduced HDL levels in mice. These findings further supported the idea of hyperhomocysteinemia being a risk factor for cardiovascular disease [10,11,12,13]. In fact, NAFLD is now considered the hepatic manifestation of the metabolic syndrome, which is sufficient to produce dyslipidaemia and increased the risk of atherosclerosis. Due to the hepatic origin, γ-glutamyltransferase (GGT) activity is intensively related to ALT activity. An observational study showed that higher GGT levels were clearly associated with hypertension among NAFLD patients [25]. Therefore, further work is required to establish the role of NAFLD in increasing cardiovascular disease.

The impact of homocysteine on hepatic steatosis was further validated by histomorphological alterations in the mice liver and Oil Red O staining of cultured primary hepatocytes. Hepatic steatosis is due to increased lipids biosynthesis and uptake or impaired lipids export and fatty acids oxidation in mitochondria. It has been demonstrated that the plasma non-esterified fatty acid pool was the primary contributor to hepatic triglycerides [26]. However, in the present study, we did not use a high-fat diet in both groups. It can therefore be assumed that other pathways play more important role in HMD-induced hepatic TG accumulation. It has been reported that hepatic DNL was elevated in NAFLD patients compared with healthy subjects [27]. The regulation of DNL in the liver involves a complex network of nuclear transcription factors and regulating enzymes. Here we found that ER membrane-bound transcription factor SREBP-1c activation was increased. The SREBP-1c dependent genes involved in triglyceride and cholesterol biosynthesis, such as ACC1α, FAS, HMG-CoAr, and FPPs were increased after Hcy treatment in primary hepatocytes. These findings indicated that increased DNL might be an important source responsible for Hcy-induced hepatic steatosis.

Another finding was that ER stress response might be involved in Hcy-induced hepatic steatosis. ER is the cellular site for sterols and lipids biosynthesis. There are three inactive transmembrane sensors which are bound to the intraluminal chaperone, glucose regulated protein GRP78/Bip. When the ER is stressed by accumulation of unfolded proteins, GRP78 is released, and the three stress sensors—i.e., activating transcription factor-6 (ATF6), inositol requiring enzyme-1α (IRE1α), and protein kinase RNA-like ER kinase (PERK)—are subsequently activated [28]. It is well recognized that some agents can cause ER stress (such as tunicamycin, TM) or ameliorate ER stress (such as 4-phyenylbutyric acid, 4-PBA). In the present study, TM treatment increased the mRNA and protein expression levels of GRP78 in primary hepatocytes. Similarly, Hcy treatment also upregulated GRP78 expression, and this effect could be reduced by 4-PBA pretreatment. These observations suggested that ER stress response might be involved in Hcy-induced hepatic steatosis. We further detected the protein levels of the activated form of ER stress sensor p-PERK and its downstream signal molecule, p-eIF2α. The results showed that p-PERK and p-eIF2α protein expression were significantly enhanced after Hcy treatment. Pretreatment with 4-PBA decreased the PERK and eIF2α phosphorylation level in hepatocytes. Since increased DNL is an important source for hepatic lipid accumulation, it is likely that the PERK-eIF2 pathway might play an important role in Hcy-induced hepatic steatosis. Under ER stress condition, phosphorylated PERK-mediated shut down protein synthesis can lead to decreased Insig-1 protein, which retains SREBP-1c precursor in the ER lumen. The subsequent translocation of SREBP-1c to Golgi leads to its activation [29]. With the activation of downstream factor eIF2, c/EBP protein is induced, which increases the expression of genes that regulate lipogenesis [30]. Taken together, the combination of enhanced lipogenesis through SREBP-1c activation contribute to ER stress-induced NAFLD [31].

## 5. Conclusions

In summary, our results are consistent with those of other studies and suggest that high methionine diet feeding increased plasma Hcy level, and the moderated hyperhomocysteinemia can promote the development of NAFLD in mice. Hcy-induced hepatic steatosis was further validated in mice primary hepatocytes. ER stress response and the PERK-eIF2α signaling pathway might be involved in the activation of transcription factor SREBP-1c and the downstream genes responsible for de novo lipogenesis.

## Figures and Tables

**Table 1:** Plasma biochemical parameters in mice.

	CT (*n* = 10)	MET (*n* = 10)
TG (mmol/L)	1.25 ± 0.10	1.66 ± 0.20 **
TC (mmol/L)	3.20 ± 0.25	3.22 ± 0.46
ALT (U/L)	30.97 ± 6.2	34.78 ± 9.8
AST (U/L)	148.98 ± 40.46	158.29 ± 19.52 *
AST/ALT	4.31 ± 0.32	6.50 ± 0.98 ***
LDL (mmol/L)	0.45 ± 0.06	0.37 ± 0.06
HDL (mmol/L)	2.49 ± 0.27	1.93 ± 0.14 ***

Results are expressed as mean ± SD. * *p* < 0.05, ** *p* < 0.015, and *** *p* < 0.001 vs. CT group. CT: standard chow diet; MET: diet supplemented with 2% methionine; TG: triglyceride; TC: total cholesterol; ALT: alanine aminotransferase; AST: aspartate aminotransferase; LDL: low-density lipoprotein cholesterol; HDL: high-density lipoprotein cholesterol.
